# Field Evaluation of Two Different Treatment Approaches and Their Ability to Control Fleas and Prevent Canine Leishmaniosis in a Highly Endemic Area

**DOI:** 10.1371/journal.pntd.0004987

**Published:** 2016-09-15

**Authors:** Emanuele Brianti, Ettore Napoli, Gabriella Gaglio, Luigi Falsone, Salvatore Giannetto, Fabrizio Solari Basano, Roberto Nazzari, Maria Stefania Latrofa, Giada Annoscia, Viviana Domenica Tarallo, Dorothee Stanneck, Filipe Dantas-Torres, Domenico Otranto

**Affiliations:** 1 Dipartimento di Scienze Veterinarie, Università degli Studi di Messina, Messina, Italy; 2 Arcoblu s.r.l., Milano, Italy; 3 Dipartimento di Medicina Veterinaria, Università degli Studi di Bari, Bari, Italy; 4 Bayer Animal Health GmbH, Leverkusen, Germany; 5 Centro de Pesquisas Aggeu Magalhães, Fundação Oswaldo Cruz, Recife, Brazil; Hebrew University, ISRAEL

## Abstract

This study investigated the efficacy of two collars for the treatment and prevention of flea infestations. Additionally the effect of these collars on the incidence of *Leishmania infantum* infection as compared with a group of vaccinated dogs was evaluated. A total of 224 young dogs from private animal shelters were enrolled in April/May into four groups: G1, 55 dogs treated with 10% imidacloprid + 4.5% flumethrin collar (Seresto, Bayer Animal Health); G2, 60 dogs treated with 4% deltamethrin collar (Scalibor protector band, MSD Animal Health); G3, 54 dogs vaccinated with CaniLeish (Virbac Animal Health); and G4, 55 dogs left non-treated as controls. Dogs were followed up at days 120 (September), 210 (December), and 360 (April-May). At those time points, clinical assessments, ectoparasite counts and blood, bone marrow and skin samples, to detect the presence of *L*. *infantum*, were performed. The efficacy of Seresto in protecting dogs from flea infestation was 100% (P < 0.01) on day 120 and 210, while animals treated with Scalibor showed a prevalence of the infestation ranging from 23.3% to 33.3% on day 120 and 210, respectively. At the end of the study, the incidence of *L*. *infantum* infection in collared dogs—based on animals being positive in any of the tests—was 5.5% in Seresto-treated dogs and 20% in Scalibor-treated dogs, resulting in overall efficacy of prevention of 88.3% for Seresto and 61.8% for Scalibor. No statistical difference was detected in *L*. *infantum* positive dogs for bone marrow PCR and/or cytology at day 360 between the CaniLeish (15.4%) and non-treated control dogs (10.0%). Both collars proved to be effective (P < 0.01) in preventing *L*. *infantum* infection throughout one transmission season, whereas no significant difference was recorded in the frequency of active infections between dogs vaccinated with CaniLeish and control dogs, emphasizing the importance of using repellent/insecticide actives as a priority measure for protection against canine leishmaniosis.

## Author Summary

Dogs are exposed to ectoparasites (e.g. ticks and fleas) and associated vector-borne infections. Among others, *Leishmania infantum* is a widespread protozoan of public health concern transmitted by phlebotomine sand flies. The prevention of canine leishmaniosis has become a priority in many endemic areas and it includes the adoption of control strategies by preventing the infection (avoiding the vector bites) or by preventing disease through vaccination. We investigated the efficacy of two collars for the treatment and prevention of flea infestations. Also, the effect of these collars on the incidence of *L*. *infantum* infection as compared with a group of vaccinated dogs was evaluated. At the end of the study, after one transmission season, both collars proved to be effective in preventing *L*. *infantum* infection, though to different levels of efficacy. However, no significant difference was recorded in the frequency of active infections between vaccinated and control dogs. Results emphasize the importance of using repellents/insecticides as a priority measure for protection against canine leishmaniosis, while vaccination can be considered as part of an integrate control program and cannot replace anti-vectorial measures.

## Introduction

The veterinary importance of ectoparasites (e.g. ticks and fleas) is characterized by their impact on the health of companion animals [1]. Ectoparasites interact intensively with their animal hosts through blood feeding, and have the capacity to transmit pathogens of both medical and veterinary significance, causing the so-called vector-borne diseases (VBD), which are among the principal causes of morbidity and mortality in companion animals [2]. In Mediterranean countries, such as Italy, ticks and fleas represent a year-round hazard especially in sheltered animals [3,4]. The control of ectoparasites in dogs, by means of ectoparasiticide products, has proved to be successful under different environmental and housing conditions [5] and efficient to reduce the risk of transmission of several VBD [6].

Visceral leishmaniosis caused by *Leishmania infantum* is a vector-borne parasitic disease affecting mainly dogs and humans [7], being endemic in southern Europe, Middle East, Central Asia and South America [8]. Dogs represent the principal reservoirs of the infection and thus play an important role in the epidemiology of the disease [7]. Canine leishmaniosis (CanL) may evolve through a plethora of clinical presentations spanning from subclinical infections to fatal illness [9]. The main method for preventing *L*. *infantum* infections in animals and humans is to avoid the bites of phlebotomine sand fly vectors by means of repellents [10,11]. Indeed, pyrethroids, either applied as spot-on formulations or as collars, have been proven effective in preventing phlebotomine sand fly bites under laboratory conditions or *L*. *infantum* infection in dogs under field conditions [11]. For example, a collar containing 4% deltamethrin (Scalibor protector band, MSD Animal Health) showed to be useful in controlling the infection by *L*. *infantum* in endemic areas with a range of efficacy from 50% to 84% after one transmission season [12,13]. A polymer matrix collar containing a combination of 10% imidacloprid and 4.5% flumethrin (Seresto, Bayer Animal Health), recently licensed for the control of ticks and fleas in dogs and cats up to eight months [14], though not registered against phlebotomine sand flies, was effective (i.e. efficacy from 93.4 to 100%) in protecting sheltered dogs living in CanL endemic areas [15,16].

In addition, considerable efforts have been put into the development of a vaccine against CanL by selecting several vaccine candidates and adjuvants, which lead to the launching of three vaccines in the past 10 years [11]. For example, a vaccine based on excretory-secretory antigens of *L*. *infantum* with *Quillaja saponaria* (LiESP-QA-21) as adjuvant, has been licensed in Europe (CaniLeish, Virbac Animal Health). Following a primary course consisting of three injections at 21-days intervals, this vaccine induces a one-year Th1-dominated cell-mediated immune response against *L*. *infantum*, protecting dogs from developing clinical signs after *L*. *infantum* infection [17,18]. When tested in the field in naïve dogs (n = 41), this vaccine showed an efficacy in preventing active infection of 68.4% and a protection against the development of clinical signs of 92.7% [19]. As none of the currently available vaccines are capable to protect against infection [20], their use must be considered as part of an integrate control program for CanL and cannot replace anti-vectorial measures.

In this study, we investigated the efficacy of two collars for the treatment and prevention of ectoparasite infestations as compared with an untreated control group. Additionally, we assessed the effect of these collars on the incidence of CanL as compared with a group of CaniLeish-vaccinated dogs.

## Methods

### Ethical statement

This was a negative controlled, multicentre study conducted according to the principles of Good Clinical Practices (VICH GL9 GCP) [21], and the Guideline on Statistical Principles for Clinical Trials for Veterinary Medicinal Products (CVMP EMA/CVMP/EWP/81976/2010) [22]. The study was performed under the framework of a large research project for monitoring and controlling vector-borne diseases and ectoparasites (including phlebotomine sand flies) in sheltered dogs. The project and activities were defined in a master agreement between the Department of Veterinary Sciences of the University of Messina and the four shelters where the study was carried out. The study protocol was approved by the Ethical Committee of the Department of Veterinary Sciences of the University of Messina (no. 002/2016, prot. 18894, March 23^rd^ 2016).

### Study sites, animals and design

Animals were housed in four private animal shelters one in Catania province (S1) and three in Syracuse province (S2-S4), Sicily (southern Italy). Study sites had a history of ectoparasite infections on dogs and were located in a *L*. *infantum* hyper-endemic area in which a mean annual incidence of *L*. *infantum* infection of 39.4% has been estimated in unprotected sheltered dogs [16] and where competent phlebotomine sand fly vectors—i.e. *Phlebotomus neglectus*, *Phlebotomus perniciosus* and *Phlebotomus perfiliewi*–occurred from late spring to autumn, i.e. May to November [16,23].

Animals at the study sites (i.e. n = 380, n = 450, n = 400 and n = 470 dogs in S1, S2, S3 and S4, respectively) were housed in open enclosures according to the time of admission into the facility, their attitude and behaviour. Dogs had a covered resting area with concrete floor with beds and an external uncovered area with concrete (S4) or fine gravel floor (S1, S2 and S3). Covered areas were separated by walls or aluminium composite panels.

In April-May 2013, a total of 247 dogs (i.e. S1 = 60, S2 = 65, S3 = 60, S4 = 62) were examined and sampled for the study enrolment (Day 0). In order to minimise the risk to include *L*. *infantum* infected dogs, only dogs with a maximum age of 18 months were selected for the study. Dogs were physically examined and weighed, and blood, skin and bone marrow samples were collected (see below). Animals were enrolled in the study if they fulfilled the following criteria: normal general health, ≥ 7 weeks to 18 months of age, not treated with ectoparasiticides within the time of activity reported for the used product and not treated with immunosuppressive drugs within 14 days prior to study start. Only dogs that tested negative for *L*. *infantum* in serology (IFAT) and PCR in skin and bone marrow at the time of inclusion were maintained in the study. Dogs included were identified using microchips and assigned to one of the four groups using a random treatment allocation plan.

Dogs in group 1 were treated with Seresto, those in group 2 with Scalibor and animals in group 3 were vaccinated with three doses (at 21-days intervals) of CaniLeish, after being tested negative with Speed Leish K (Virbac). Also, according to the requirements for CaniLeish vaccination, only dogs older than 6 months were included in that specific group. Group 4-dogs were kept as non-treated controls. Within the study sites dogs included were kept in pens in smaller groups with an average size of 6 (1 to15) dogs per pen. Randomization was conducted pen-wise in order to avoid animals from different groups being in direct physical contact and pens containing study animals were patchily disseminated within the study site. Collared dogs (groups 1 and 2) were kept under label-conform medication for approximately seven months, according to the length of *L*. *infantum* transmission season in the study area.

Dogs were followed up on days 120 ±10 (September), 210 ±10 (December) and 360 ±15 (April-May) after inclusion. At each follow-up, dogs were physically examined for ectoparasite (flea and tick) presence and CanL related signs were recorded (e.g. loss of weight, dry exfoliative dermatitis, muscular atrophy, periocular alopecia, pale mucous membranes, onychogryphosis, lymphadenopathy, splenomegaly and conjunctivitis). During those follow up visits skin and blood samples were also collected. Briefly, blood samples of approximately 5 ml were collected in serum separator gel tubes (Vacumed) from the brachial or jugular veins, being immediately refrigerated (+4°C). Skin tissue samples (about 0.5 cm²) were collected from the inter-scapular region and stored in individual micro-tubes containing 1 ml of phosphate buffered saline (PBS) solution. Additionally, bone marrow samples were collected at the enrolment and on days 210 ±10 and 360 ±15. Bone marrow samples were aspirated from the iliac crest using Rosenthal needles (16 or 18 gauge), then a few drops were smeared on slides for cytological examination and the remaining part was stored in individual micro-tubes with 1 ml of PBS solution. Dogs included in the collar treated groups were wearing collars up to day 210 of the study. Seresto collars were replaced only if they were lost or if the animal’s weight increased above the threshold of 8 kg for the small collar size, whereas Scalibor protector-bands were replaced in case of losses and substituted on day 120 according to the recommendations given in the product leaflet.

All dogs included in the study were observed daily for any changes in their health and abnormal health conditions were recorded. The use of other ectoparasiticides on dogs or in the environment was not allowed throughout the study period. However for all groups, individual treatments with fipronil in spot-on formulation were eventually authorized when heavy tick or flea infestations occurred. Personnel performing laboratory tests was blinded.

### Diagnostic procedures

In the laboratory, blood samples were centrifuged (1,500 *g* for 10 minutes) and the serum was split into two aliquots. Serum, skin and bone marrow samples were stored at –20°C. Serum samples were tested for circulating anti-*L*. *infantum* antibodies by IFAT using a cut-off of 1:80 as described elsewhere [24]. Positive sera were also titrated using serial dilutions until negative. DNA extraction and PCR amplification of *Leishmania* kinetoplast DNA was performed on bone marrow and skin samples as described elsewhere [16]. Bone marrow smears were stained with MGG Quick Stain (Bio Optica, Italy) and microscopically examined for *L*. *infantum* amastigotes. Each smear was examined for about 10 minutes under light microscopy (100 microscopic fields) using a 100X oil immersion objective.

Dogs in the two collar treated groups and non-treated control dogs were defined as infected by *L*. *infantum* when positive in at least one of the diagnostic methods (i.e. IFAT, PCR on skin and bone marrow, and cytology) during the course of the study. Since the presence of anti-*L*. *infantum* antibodies in CaniLeish-vaccinated dogs could be due to the immune response induced by the vaccine, the detection of the parasite in bone marrow samples by PCR and/or cytology at day 210 and 360 was considered as indicative of a failure in controlling the infection. At the last visit, infections were further classified as active infections when IFAT positive results (≥ 1:160) were associated with bone marrow PCR and cytology positive findings; dogs with active infections were ranked into sick or clinically healthy according to the presence of clinical signs [9].

### Entomological survey

At each site, light traps were used to collect phlebotomine sand flies. Starting from May 2013, two traps were placed biweekly in each shelter at 50 cm above the ground before sunset and left *in situ* for at least 12 hours (i.e. from 6:00 p.m. to 6:00 a.m.). Monitoring activity was suspended in November 2013 after three consecutive negative trapping sessions. Phlebotomine sand flies captured were separated from other insects, differentiated by sex with the aid of a stereomicroscope and stored into single vials containing 70% ethanol according to site and sampling date. Each sand fly specimen was prepared for microscopic observation as described elsewhere [23] and identified at species level using appropriate morphological keys [25].

### Statistical analysis

The minimum sample size of 48 dogs per group was determined considering an expected *L*. *infantum* incidence of 4% in vector protected (collared) dogs (Seresto and Scalibor groups) and of 16% in animals exposed to vector bites (CaniLeish and control groups) with a power of 85% and 95% confidence level [26]. The homogeneity for dog variables such as sex, age, coat length and body weight of the four groups was calculated at the inclusion (Day 0) using χ^2^ test or Fisher’s Exact test for qualitative data (sex, coat length) and using analysis of variance (ANOVA).

Efficacy (%) in preventing flea infestation was calculated using the following formula: Efficacy = (% of infested animals in control group—% of infested animals in treatment group)/(% of infested animals in control group) x 100. *Leishmania infantum* incidence for each group was calculated as year-crude incidence (YCI) considering only results of the final sampling (day 360) regardless of what happened in between as follows: Year crude incidence = number of *L*. *infantum* newly infected animals/(number of negative animals initially enrolled − number of animals lost to follow up) × 100. In addition, in order to overcome the problem of dogs lost to follow-up during the study, the incidence of *L*. *infantum* infection was studied using the incidence density rate (IDR) [27], adapted on a monthly basis using a standard 30 days/month. IDRs were calculated at each follow-up as the number of positive dogs, either serologically or molecularly, divided by the number of dog-months of follow-up (i.e. the number of months between the previous and the current assessment for each dog at risk for *L*. *infantum* infection). IDRs were expressed per year. Dogs tested once (e.g. lost, dead) did not contribute at any time to the incidence calculation.

The efficacy (%) in preventing *L*. *infantum* infection was calculated per each collar treated group using the same formula adopted to calculate the efficacy against flea infestation. The significance of the efficacies was tested using χ^2^ test.

Differences in the frequency of bone marrow PCR and cytology results as well as in the number of active infections between vaccinated and untreated dogs were analysed using χ^2^ test or Fischer’s test, as appropriate. The level of significance was set at 0.05.

## Results

The four groups were homogenous (P > 0.05) for variables at the time of inclusion with the exception of age and weight of animals in CaniLeish group because the label of this vaccine requires a minimum age of 6 months. Of the 247 dogs initially screened, 23 were excluded because they were either positive at IFAT or at IFAT and cytology (n = 2), exceeded the maximum age defined in the inclusion criteria (n = 4) or died before the first follow-up (n = 16). Additionally one dog was adopted. During the study, collars were replaced on 13 dogs in the Seresto treated group to readjust the dose on the basis of the changes in weight and/or were replaced one or more times for loss or damages in 21 and 36 dogs of Seresto and Scalibor groups, respectively.

The efficacy of the Seresto against flea infestation was 100% (P < 0.01) on days 120 and 210, whereas animals treated with Scalibor showed a prevalence of flea infestation of 23.3% and 33.3% on days 120 and 210, respectively (Table 1).

**Table 1:** Presence of flea and tick infestation at the different times of the study.

Groups	Day of the study (month/s)		
	0 (April/May 2013)	120 (September 2013)	210 (December 2013)
**G1** (Seresto)			
N. of examined dogs	55	55	55
N. of flea infested dogs	13 (23.6%)	0	0
N. of tick infested dogs	0	0	0
**G2** (Scalibor)			
N. of examined dogs	60	60	60
N. of flea infested dogs	6 (10.0%)	14 (23.3%)	20 (33.3%)
N. of tick infested dogs	0	2 (3.3%)	0
**G3** (CaniLeish)*			
N. of examined dogs	54	54	53
N. of flea infested dogs	12 (22.2%)	12 (22.2%)	19 (35.9%)
N. of tick infested dogs	2 (3.7%)	0	0
**G4** (Control)			
N. of examined dogs	55	55	51
N. of flea infested dogs	1 (1.8%)	12 (21.8%)	14 (27.5%)
N. of tick infested dogs	0	1 (1.8%)	0

*As CaniLeish vaccine is not expected to have any ectoparasiticide efficacy, results against fleas in this group can be seen as that of an additional non-treated control

Additionally, between days 120 and 210, 24 dogs (11 in the Scalibor group, 8 in the CaniLeish group and 5 in the control group) were found heavily infested by fleas and received an individual rescue treatment with a spot-on product containing fipronil. Tick infestations were only very sporadically observed in dogs throughout the study (Table 1), and this did not allow any meaningful statistical evaluation of the efficacy of the two collars against ticks.

The number of dogs positive for *L*. *infantum* at any test and at any time point varied from four in the Seresto group to 35 in the CaniLeish group (Table 2).

**Table 2:** Serological, molecular and cytology results only of dogs positive for *Leishmania infantum* at IFAT, PCR on skin or bone marrow (BM) or BM cytology at any time point during the study.

Group	Site and code	Day 120 (September 2013)		Day 210 (December 2013)			Day 360 (April-May 2014)				
		IFAT	Skin PCR	IFAT	Skin PCR	BM PCR	IFAT	Skin PCR	BM PCR	BM Cytology	Clinical signs
**G1**	S1021	1:80	-	-	-	-	-	-	-	-	-
	S1028	-	-	1:80	-	-	1:80	-	-	-	Ln
	S2020	-	-	1:80	-	-	1:80	+	-	-	-
	S2025	-	+	-	-	-	-	+	-	-	-
**G2**	S1044	-	-	-	-	-	1:80	+	-	-	-
	S1052	-	-	-	-	-	1:1280	-	+	-	Ln, De
	S1059	-	-	-	-	-	1:80	-	-	-	-
	S1063	-	-	-	-	-	-	-	+	-	-
	S2051	-	+	-	-	-	-	+	-	-	-
	S2067	-	+	-	+	-	1:80	-	-	-	Ln
	S3047	-	-	-	-	-	1:80	-	-	-	-
	S3065	-	-	-	-	-	-	+	-	-	-
	S3067	-	+	-	-	-	1:80	-	+	-	-
	S4042	-	-	-	-	-	1:80	-	-	-	-
	S4043	-	-	-	-	-	1:80	-	-	-	-
	S4044	1:80	-	-	-	-	-	-	-	-	-
	S4046	-	-	1:80	-	-	1:160	-	-	+	-
	S4048	-	-	-	+	-	-	-	-	-	-
**G3**	S1002	-	-	-	-	-	1:80	+	-	-	Ln
	S1003	-	-	1:80	-	-	1:80	-	-	-	Ln
	S1005	-	+	1:80	-	+	1:80	-	-	-	Ln
	S1006	-	-	-	-	-	1:1280	-	+	-	-
	S1008	-	+	-	-	-	-	-	-	-	-
	S1013	-	+	-	-	-	-	-	+	-	-
	S2007	1:80	-	-	+	-	1:80	-	-	-	-
	S2010	-	-	-	+	-	-	+	-	-	-
	S2011	1:80	-	1:160	-	-	1:1280	+	+	-	-
	S2014	-	+	-	-	-	1:80	-	-	-	-
	S2015	1:80	-	1:160	-	-	1:160	-	-	-	-
	S2016	-	+	-	+	-	1:1280	-	-	-	-
	S2017	1:80	-	1:160	+	+	1:2560	+	+	+	Ln
	S2018	-	-	-	-	-	1:80	+	-	-	-
	S3003	-	-	-	-	-	1:80	-	-	-	Ln, Wl
	S3004	-	+	1:80	-	-	1:80	-	-	+	-
	S3005	-	-	-	-	-	1:80	-	-	-	Ln, De
	S3006	-	-	1:80	-	+	1:320	-	+	+	Ln
	S3007	-	-	-	-	-	1:80	-	-	-	-
	S3008	-	-	-	-	-	1:80	-	-	-	-
	S3010	-	-	-	-	-	1:80	-	-	-	Ln
	S3011	-	-	-	-	-	1:80	-	-	-	-
	S3012	-	-	1:80	-	-	1:80	-	-	-	Ln
	S3013	-	-	1:80	-	-	1:80	-	-	-	Ln
	S3014	-	+	-	-	-	1:80	-	-	-	
	S4001	-	-	-	+	-	-	+	-	-	
	S4002	-	-	-	-	-	1:80	+	-	-	Ln
	S4004	-	+	-	+	-	1:80	-	+	-	Ln
	S4005	-	+	-	-	-	1:80	+	-	-	Ln
	S4006	-	-	-	+	-	1:80	-	-	-	
	S4007	-	-	-	-	-	1:80	+	-	-	
	S4009	-	-	-	-	-	1:80	-	-	-	
	S4010	-	-	-	-	-	1:80	-	-	-	
	S4013	-	-	-	+	-	-	-	-	-	
	S4049	-	-	-	-	-	1:80	-	-	+	
**G4**	S1030	-	+	-	-	-	-	-	-	-	Ln
	S1032	-	+	-	-	-	1:80	+	-	-	Ln
	S1033	-	+	-	-	-	-	+	-	-	
	S1034	-	-	-	-	-	1:1280	-	+	-	
	S1035	-	+	1:80	-	-	1:80	+	-	+	Ln
	S1036	-	+	1:80	-	-	-	+	-	-	
	S1037	-	+	1:80	-	+	1:80	+	-	-	
	S1039	-	+	1:80	+	-	1:80	+	-	-	Ln
	S1041	-	-	1:80	-	-	1:320	-	+	-	
	S1043	-	-	1:80	-	-	-	-	-	+	
	S2056	-	+	1:80	-	-	1:80	-	-	+	
	S2058	-	-	1:80	+	-	1:80	-	-	-	
	S2059	-	-	-	-	-	1:1280	-	-	-	
	S3035	-	+	-	-	-	-	-	-	-	
	S3036	-	-	-	-	-	1:80	-	-	-	
	S3052	-	-	1:80	-	-	-	-	-	-	
	S3053	-	-	1:80	-	-	1:80	-	-	-	
	S3054	-	+	-	-	+	-	-	-	-	Ln
	S3055	-	-	1:80	-	-	1:80	-	-	-	
	S3057	-	-	1:80	+	+	1:640	-	-	-	
	S3060	-	+	-	-	-	-	-	-	-	
	S3062	-	-	1:80	-	-	1:640	-	-	-	
	S3063	-	+	-	-	-	-	-	-	-	
	S4050	-	-	1:80	+	-	1:160	-	-	-	
	S4055	-	-	1:80	-	-	1:160	+	-	-	Ln
	S4056	-	-	-	-	-	1:80	+	-	-	

Legend: De = dry-exfoliative dermatitis; Ln = lymph node enlargement; Wl = weight loss.

At the last visit, three dogs in the Seresto group and 12 dogs in Scalibor group tested positive for *L*. *infantum* in at least one of the diagnostic tests, with the IFAT being the test with the highest number of positive animals (Table 2).

The YCI calculated on the total amount of dogs remained in the study until the last visit was 5.5% (3/55), 20% (12/60) and 38% (19/50) in the Seresto, Scalibor and control groups, respectively, with a statistical significant difference between Seresto *vs*. controls (P < 0.001) and Scalibor *vs*. controls (P < 0.05). Accordingly, the mean IDR ranged from 7.8% (Seresto) to 66.9% (Controls) (Table 3), resulting in an overall efficacy of the two collars in preventing *L*. *infantum* infection of 88.3% in the Seresto group and of 61.8% in the Scalibor group (P < 0.01).

**Table 3:** Incidence Density Rate (IDR) in the study population.

Group/Phase	Cohort dogs (negative at previous sampling)	New cases	Dog-months	IDR/ month	New cases	Dog-years	IDR/year
**G1** (Seresto)							
Baseline	55						
Follow-up 1	55	2	224.95	0.89	2	18.75	10.67
Follow-up 2	53	2	156.35	1.28	2	13.03	15.35
Follow-up 3	51	0	231.54	0.00	0	19.30	0.00
Total		4	612.84	0.65	4	51.07	**7.83**
**G2** (Scalibor)							
Baseline	60						
Follow-up 1	60	4	245.4	1.63	4	20.45	19.56
Follow-up 2	56	2	165.76	1.21	2	13.81	14.48
Follow-up 3	54	8	245.16	3.26	8	20.43	39.16
Total		14	656.32	2.13	14	54.69	**25.60**
**G4** (Control)							
Baseline	55						
Follow-up 1	55	12	224.95	5.33	12	18.75	64.01
Follow-up 2	39	10	115.05	8.69	10	9.59	104.30
Follow-up 3	28	4	126.00	3.17	4	10.50	38.10
Total		26	466.00	5.58	26	38.83	**66.95**

An equal number of three dogs scored positive at bone marrow PCR in the CaniLeish group (5.7%) and control group (5.9%) at day 210, whereas no statistical difference (P = 0.417) was detected in animals positive at bone marrow PCR and/or cytology at day 360 in the CaniLeish group (15.4%; 8/52) and the control group (10%; 5/50). The majority of these positive animals was also positive to IFAT, with titres ranging from 1:180 to 1:2,560 in the CaniLeish group, and from 1:80 to 1:1,280 in the control group (Table 2). Active symptomatic infections, characterized by high IFAT titres (i.e. 1:320 and 1:2,560), positive PCR and cytological results associated to lymph node enlargement, were diagnosed in two dogs of the CaniLeish group (Table 2), but no differences in the frequencies of such events were found between the CaniLeish vaccinated and the control group (P = 0.495).

Phlebotomine sand flies (n = 2,008), belonging to six species, were trapped from the end of May (S1) up to October (at all sites). The largest number of phlebotomine sand flies was captured in S3 (n = 910) followed by S1 (n = 733), S4 (n = 256) and S2 (n = 109). The largest variability of species (n = 6) was found in S1 where the most prevalent species were *P*. *perniciosus* (n = 521) and *P*. *perfiliewi* (n = 124). *Sergentomyia minuta* and *P*. *perniciosus* were the most common species in all the sites with frequencies ranging from 11.3% (S1) to 95.1% (S3) and from 4.9% (S3) to 71% (S1), respectively. *Phlebotomus perniciosus* was systematically captured in all the trapping sessions, but not at the end of July in S2. *Phlebotomus sergenti* (one male and one female) and *P*. *papatasi* (one male) were captured in site S1 only in June-July and July, respectively, while *P*. *neglectus* was only captured in S4 in June.

No abnormalities were observed in collared dogs in the seven months of application of the collars or as a consequence of the vaccination, except three dogs in the Scalibor treated group showing neck cutaneous lesions, appearing two to five months after collar application, likely due to the mechanical scrubbing of the area with the collar. All lesions recovered from 3 to 25 days after further slackening of the Scalibor without any treatment (1) or following topical treatment with antibiotic cream or antibacteric foam (2). Five dogs (one from the Seresto group and four from the control group) were affected by demodectic mange and one dog from the control group was affected by sarcoptic mange. They were treated topically with 25 ml/5l amitraz once every 4 days for 20 days.

## Discussion

Fleas were the most prevalent ectoparasites found in this trial, whereas ticks were only sporadically found, most likely due to the prophylactic measures (e.g. isolation and preventive treatment of dogs at their first entrance in the shelters) put in place on all the study sites that have resulted in a satisfactory control of ticks.

A significant difference in the efficacy against fleas was found between the two investigated collars. While no fleas were found in the Seresto treated animals at any of the post-treatment follow up time points, an increasing percentage of the Scalibor treated animals were flea infested at days 120 (23.3%) and 210 (33.3%), which was actually similar to those in the control animals.

The high efficacy of the Seresto in protecting dogs against fleas was already documented in other field trials conducted with larger number of dogs and exposed to high ectoparasite burdens [4,28], whereas for Scalibor only laboratory studies were conducted against fleas and ticks [29–32].

In spite of the different natures and aims of the intervention approaches against *L*. *infantum* used in this study (i.e. preventing infection or disease development), their effectiveness was assessed at the same time using the same diagnostic procedures in animals living closely together. The study was conducted in a hyper-endemic area for CanL, as indicated by the high rate of exposure (YCI = 38%; IDR = 66.95%) in control dogs. As expected, the two approaches (collars and vaccine) showed different results in the prevention of *L*. *infantum* infection, which are intimately linked to their different modes of action: reducing the risk of infection versus reducing the risk of disease progression. Indeed, both collars proved to be significantly effective (p<0.01) in preventing *L*. *infantum* infection with an overall efficacy of 88.3% in the Seresto group and 61.8% in the Scalibor group. All the positive animals in the Seresto group and the majority in the Scalibor group (77.8%) presented low IFAT titres (1:80) with only a few in the Scalibor group scoring positive also to bone marrow PCR or to cytology, but without evidence of active disease.

In the CaniLeish group, which remained unprotected against phlebotomine sand fly bites, the number of dogs positive for *L*. *infantum* at PCR or cytology was significantly higher as compared to the Seresto and Scalibor groups. The detection of *L*. *infantum* in tissues such as bone marrow either by PCR and/or cytology (Table 2) is indicative for the parasite dissemination from the site of entry (i.e. skin). This diagnostic finding is also suggestive for an ineffective immune response against the infection [19,33] as indicated by the finding of two sick dogs in the CaniLeish group at day 360. Also, the finding of five IFAT-positive dogs in the vaccinated group with high titres (1:320–1:2,560) was indicative of active infection. In fact, IFAT may become positive in CaniLeish animals [17] but vaccination-induced seroconversion usually occurs within 8–12 weeks after vaccination [18]. Conversely the majority of IFAT positive dogs in group 3 seroconverted several months after vaccination suggesting a response to natural *L*. *infantum* infection rather than a serocoversion induced by the vaccination. Interestingly, in a previous field trial conducted with naïve dogs in a CanL endemic area, a total of one and five active infections were observed in a group of 41 vaccinated animals after the first and second year, respectively [19]. The number of active infections herein reported in the CaniLeish group (2/52; 3.8%) is not significantly different neither from that recorded in [19], nor from that observed in control animals of this study. This lack of difference between the CaniLeish and the control group could be due to the number of animals not being enough for distinguishing this specific question, as well as to the period of observation of animals (i.e. one year only). As a matter of fact, also in a previously reported field trial no significant differences in *L*. *infantum* infection were found between vaccinated (41 dogs) and control (39 dogs) groups at one-year follow-up [19]. Finally, the presence of IFAT detectable antibodies for long periods in vaccinated dogs poses practical issues regarding the use of serological assays to screen animals in CanL-endemic areas.

The entomological survey confirmed the presence of proven phlebotomine sand fly vectors in all study sites, being their period of activity from late May up to October, which is in agreement with previous studies in southern Italy [16,23,25] and indicates that the *L*. *infantum* transmission season lasts about 6 months in the study area. Although *P*. *perniciosus* and *S*. *minuta* were the most frequent species in all study sites, the species composition and relative frequencies varied among shelters. However, it is important to emphasize that the entomological survey herein carried out aimed to assess the phlebotomine sand fly fauna in the four study sites, but not to estimate their abundance. The retrieval of *P*. *sergenti* confirms previous reports in the eastern part of Sicily [23,34]. This sand fly species is involved in the transmission of *Leishmania tropica*, the agent of anthroponotic cutaneous leishmaniosis in the Middle East and Africa, which, however, has the rock hyrax (*Procavia capensis*) as a reservoir host in Israel, Jordan and the Palestinian Authority [35,36].

### Concluding remarks

The Seresto collar proved to be effective in protecting dogs against flea infestation, while no difference in the rate of infestation was observed between animals treated with Scalibor and non-treated dogs. Both collars were efficacious in preventing *L*. *infantum* infection, with efficacies ranging from 61.8% for Scalibor to 88.3% for Seresto after one transmission season. The frequency of active infections in dogs vaccinated with CaniLeish was similar to that of a previous field trial [19], and no significant differences in *L*. *infantum* infection rates were recorded between vaccinated and controls animals after one year. All the products proved to be safe and their use should be considered when control strategies against CanL are planned. However, because of its inefficacy in the prevention of *L*. *infantum* infection and according to the company prescriptions, the vaccine is always recommended in combination with repellents/insecticides and cannot replace their use in CanL endemic areas.
